# Oncological outcomes in patients with stage I testicular seminoma and nonseminoma: pathological risk factors for relapse and feasibility of surveillance after orchiectomy

**DOI:** 10.1186/1746-1596-8-57

**Published:** 2013-04-08

**Authors:** Kazuhiro Kobayashi, Toshihiro Saito, Yasuo Kitamura, Tomohiro Nobushita, Takashi Kawasaki, Noboru Hara, Kota Takahashi

**Affiliations:** 1 Department of Urology, Niigata Cancer Center Hospital, Kawagishi-cho 2, Niigata 951-8566, Japan; 2 Department of Pathology, Niigata Cancer Center Hospital, Kawagishi-cho 2, Niigata 951-8133, Japan; 3 Division of Urology, Department of Regenerative and Transplant Medicine, Graduate School of Medical and Dental Sciences, Niigata University, Asahimachi 1, Niigata 951-8510, Japan; 4 Division of Molecular Oncology, Department of Signal Transduction Research, Graduate School of Medical and Dental Sciences, Niigata University, Asahimachi 1, Niigata, 951-8510, Japan

**Keywords:** Stage I seminoma, Stage I nonseminoma, Surveillance, Outcome

## Abstract

**Background:**

Surveillance after orchiectomy has recently been a management option in patients with stage I seminoma, while it remains controversial in those with stage I nonseminoma, and the risk factor associated with relapse is still a matter of concern in both entities. This study was performed to explore pathological risk factors for post-orchiectomy relapse in patients with stage I seminoma and nonseminoma, and to assess oncological outcomes in those managed with surveillance.

**Methods:**

In this single institution study, 118 and 40 consecutive patients with stage I seminoma and nonseminoma were reviewed, respectively. Of the 118 patients with stage I seminoma, 56 and one received adjuvant radiotherapy and chemotherapy, respectively, and 61 were managed with surveillance. Of the 40 men with stage I nonseminoma, 4 underwent adjuvant chemotherapy and 36 were managed with surveillance.

**Results:**

No patient had cause-specific death during the mean observation period of 104 and 99 months in men with seminoma and nonseminoma, respectively. In men with stage I seminoma, 1 (1.7%) receiving radiotherapy and 4 (6.6%) men managed with surveillance had disease relapse; the 10-year relapse-free survival (RFS) rate was 93.4% in men managed with surveillance, and their RFS was not different from that in patients receiving adjuvant radiotherapy (logrank *P*=0.15). Patients with tunica albuginea involvement showed a poorer RFS than those without (10-year RFS rate 80.0% vs. 94.1%), although the difference was of borderline significance (*P*=0.09). In men with stage I nonseminoma, 9 (22.5%) patients experienced relapse. Patients with lymphovascular invasion seemingly had a poorer RFS than those without; 40.0% and 18.7% of the patients with and without lymphovascular invasion had disease relapse, respectively, although the difference was not significant (logrank *P*=0.17).

**Conclusion:**

In both men with stage I seminoma and nonseminoma, surveillance after orchiectomy is a feasible option. However, disease extension through tunica albuginea might be a factor associated with disease relapse in patients with organ-confined seminoma, and those with stage I nonseminoma showing lymphovascular invasion may possibly be at high risk for disease relapse.

## Background

Testicular germ cell tumor is histopathologically classified into seminoma and nonseminomas, and nonseminomas are subclassified into embryonal carcinoma, choriocarcinoma, yolk sac tumor, teratoma, and mixed germ cell tumors. After an orchiectomy, the most feasible therapeutic option is determined with risk assessment based on the pathological diagnosis and clinical staging [1]. Nonseminoma is more potent to metastasize and lead to poorer prognosis compared with seminoma at same stage. Surveillance has recently been a management option in many patients with stage I seminoma, and treatment and follow-up strategy vary according to the clinicopathological characteristics in stage I nonseminoma [2,3]. However, risk factors associated with relapse still remain controversial in both entities. This study was performed to explore pathological risk factors associated with disease relapse in men with stage I seminoma and nonseminoma, and to evaluate oncological outcomes in those managed with surveillance after orchiectomy.

## Methods

### Patients

This retrospective research project was approved by the Ethics Committee of Niigata Cancer Center Hospital. Written informed consent was obtained from all the patients. In total, 158 consecutive patients who were treated for stage I testicular germ cell tumors at the Department of Urology, Niigata Cancer Center Hospital between May 1980 and December 2008 were enrolled in the present study; 118 and 40 men were pathologically diagnosed with seminoma and nonseminomas, respectively. All of them received high orchiectomy. Disease stage was determined with abdominal-pelvic computerized tomography (CT) and thoracic CT or chest roentgenography. Patients’ characteristics were presented in Table 1; serum tumor markers such as lactate dehydrogenase (LDH), α-fetoprotein (AFP), and human chorionic gonadotropin β subunit (hCGβ) had been normalized after orchiectomy in all of them. Of the 118 patients with stage I seminoma, 56 received adjuvant radiotherapy (para-aortic with or without ipsilateral pelvic irradiation of 28.9 Gy in 17 fractions), 61 were managed with surveillance without adjuvant therapy, and one underwent adjuvant chemotherapy.

**Table 1:** Patients’ demographics at diagnosis

	**Total (n=158)**	**Seminoma (n=118)**	**Nonseminoma (n=40)**	**P value seminoma vs nonseminoma**
age [y.o.]	37.0 ± 10.6	39.0 ± 9.8	31.2 ± 11.1	<0.01*
mean ± SD (range)	(1–65)	(22–65)	(1–54)	
side n(%) right	91 (57.6%)	70 (59.3%)	21 (52.5%)	0.45
left	67 (42.4%)	48 (40.7%)	19 (47.5%)	
tumor size [cm]	6.0 ± 2.7	6.4 ± 2.8	4.8 ± 1.9	<0.01
mean ± SD (range)	(1.5–18)	(1.5–18)	(2.0–9.0)	
pT n(%) T1	48 (30.4%)	32 (27.1%)	16 (40.0%)	0.51
T2	63 (39.9%)	48 (40.7%)	15 (37.5%)	
T3	2 (1.3%)	2 (1.7%)	0 (0%)	
T4	1 (0.6%)	1 (0.8%)	0 (0%)	
Tx	44 (27.8%)	35 (29.7%)	9 (22.5%)	
LDH (IL/l)	449.8 ± 453.5	491.9 ± 504.9	323.6 ± 197.6	0.05
mean ± SD (range)	(121–3043)	(112–3043)	(121–945)	
AFP (ng/ml)	124.0 ± 801.7	2.9 ± 1.4	474.1 ± 1544.0	<0.01
mean ± SD (range)	(1.0–9363.1)	(1.0–8.2)	(2.5–9363.1)	
hCGβ (ng/ml)	1.0 ± 1.8	0.9 ± 1.8	1.2 ± 1.7	0.37
mean ± SD (range)	(<0.1–10.9)	(<0.1–10.9)	(<0.1–6.69)	

LDH, lactate dehydrogenase; AFP, α-fetoprotein, hCGβ, human chorionic gonadotropin β subunit.

Histopathological subtypes in the nonseminoma group were shown in Table 2. Rare histotypes potentially coexistent with teratoma were not described [4]. Of the 40 men with stage I nonseminomas, 4 underwent cisplatin-based systemic chemotherapy and 36 were managed surveillance without additional treatment. Follow-up protocol was principally as follows: monthly measurement of tumor markers and bimonthly or 3 monthly thoracic-abdominal-pelvic CT for the initial 6 months, 3-month interval measurement of tumor markers and thoracic-abdominal-pelvic CT for the next 2 to 3 years, and 6- to 12-month interval examinations for the next 5 to 10 years. The mean observation period was 104 (range: 19 – 340) months in the seminoma group, and it was 99 (range: 23 – 261) months in the nonseminoma group.

**Table 2:** Histological diagnosis in patients with nonseminomas (n=40)


Pure	10 (25.0%)
Embryonal carcinoma	8 (20.0%)
York sac tumor	1 (2.5%)
Teratoma	1 (2.5%)
Mixed	30 (75.0%)
Teratoma	21 (52.5%)
Embryonal carcinoma	20 (50.0%)
York sac tumor	20 (50.0%)
Seminoma	19 (47.5%)
Choriocarcinoma	7 (17.5%)

### Statistical analysis

In addition to the chi-square test for categorical variables, the Welsh-corrected *t* test was used to compare unpaired continuous parameters among subgroups. Survival curves were generated using the method of Kaplan and Meier, and they were compared using the logrank test. Hazard ratio (HR) and 95% confidence interval (CI) were calculated using the Cox proportional hazard model. Statistical analyses were calculated and tested using SPSS software version 15.0 (SPSS, Inc., Chicago, IL, USA) and Prism Version 4.02 (GraphPad software, Inc., San Diego, CA, USA) for Windows-based computers. The test was two-sided and *P*< 0.05 was considered significant.

## Results

### Outcomes of patients with stage I seminoma

Table 3 shows outcomes in the seminoma group. In 80s and 90s, 80.0% and 91.3% of the patients underwent adjuvant radiotherapy, respectively, whereas only 3.5% of them experienced it in 2000s (*P*<0.001). In men receiving radiotherapy, 1 (1.7%) had disease relapse, and in those managed with surveillance, 4 (6.6%) developed metastasis (*P*=0.19). No patient had cause-specific death in this group.

**Table 3:** Outcomes of patients with stage I seminoma

	**Radiation (n = 56)**	**Surveillance (n = 61)**	**P value**
Follow–up months			<0.01
mean ± SD (range)	174 ± 54 (16–339)	67 ± 50 (5–257)	
Era n (%)			<0.001
1980s	12 (80.0%)	3 (20.0%)	
1990s	42 (91.3%)	4 (8.7%)	
2000s	2 (3.5%)	54 (94.7%)	
Relapse			0.19
n (%)	1 (1.7%)	4 (6.6%)	
Site	chest wall 1	lymph-node 4	
		liver 1	
Time to relapse	16 months	5–7 (mean 7)	
Treatment after relapse			0.99
Chemotherapy	1 (100%)	4 (100%)	
Radiation	0 (0%)	1 (25%)	
Prognosis			
Cause-specific death	0 (0%)	0 (0%)	0.99
Death of other causes	4 (7.1%)	1 (1.6%)	0.13

One patient receiving adjuvant chemotherapy was excluded.

**Figure 1:**
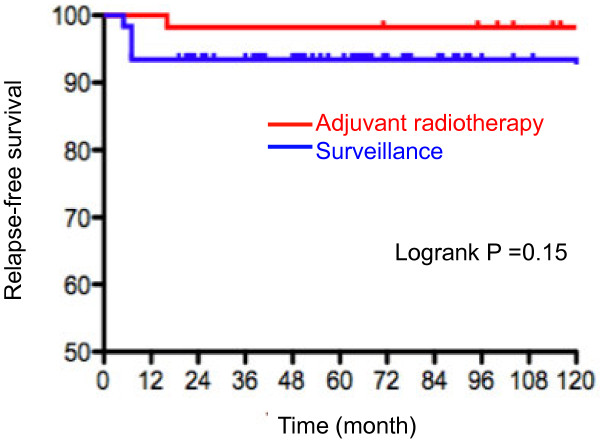
Relapse-free survival in patients with stage I seminoma receiving adjuvant radiotherapy or managed with surveillance.

**Figure 2:**
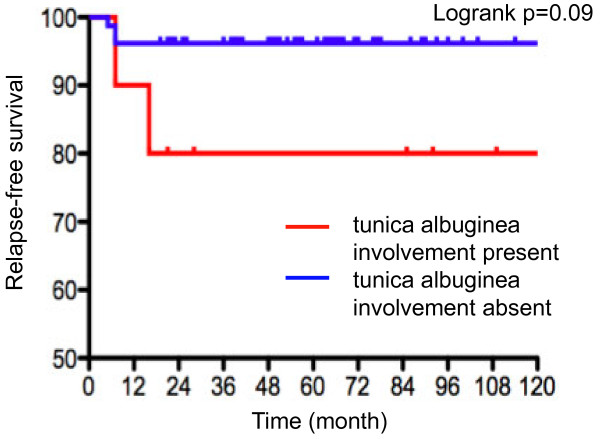
Impact of tunica albuginea involvement on relapse-free survival in patients with stage I seminoma.

Both the 5-year and 10-year relapse-free survival rates were 93.4% in men managed with surveillance, while they were 98.2% in men receiving adjuvant radiotherapy. Relapse-free survival was not different between patients managed with surveillance and receiving radiotherapy (*P*=0.15, Figure 1). We further examined the association of patients or disease characteristics with oncological outcomes (Table 4). None of the primary tumor size, serum marker levels, pT stage, and radiotherapy was associated with disease relapse. Concerning pathological characteristics, patients with tunica albuginea involvement showed a poorer relapse-free survival than those without, although the difference was of borderline significance (logrank *P*=0.09, Figure 2).

**Table 4:** Associations between disease background and relapse in patients with stage I seminoma

**Variables**	**Category**	**No.**	**Relapse**	**Univariate analyses**		
			**n (%)**	**P value**	**HR**	**95%CI**
Age	<40	70	1 (2.5%)	0.11	0.17	0.02-1.50
	≥40	48	4 (8.3%)		1	
Size	≤5 cm	45	2 (4.4%)	0.77	1.33	0.19-9.47
	>5 cm	59	2 (3.4%)		1	
βhCG	Normal	29	2 (6.9%)	0.53	1.78	0.30-10.6
	Elevated	75	3 (4.0%)		1	
LDH	Normal	48	1 (2.1%)	0.29	0.31	0.03-2.75
	Elevated	50	4 (8.0%)		1	
pT	pT1	32	2 (6.3%)	0.93	1.09	0.18-6.51
	pT2-4	51	2 (3.9%)		1	
Lymphovascular invasion	No	37	3 (8.1%)	0.41	2.11	0.35-12.6
	Yes	51	2 (3.9%)		1	
Tunica albuginea involvement	No	79	3 (3.8%)	0.07	0.19	0.03-1.13
	Yes	10	2 (20.0%)		1	
Spermatic cord invasion	No	90	5 (5.6%)	-	-	-
	Yes	3	0 (0%)			
Radiotherapy	No	61	4 (6.6%)	0.23	3.83	0.43-34.3
	Yes	56	1 (1.8%)		1	

### Outcomes of patients with stage I nonseminoma

Of the 40 men with stage I nonseminoma, 36 (90%) were managed with surveillance and 4 (10%) received adjuvant chemotherapy after orchiectomy; patients’ characteristics such as the tumor markers and size did not differ between those with surveillance and adjuvant chemotherapy (data not shown). In Table 5, outcomes of the patients with stage I nonseminomas were summarized. In this group, 9 (22.5%) patients had disease relapse. Of the patients managed with surveillance, 9 (25.0%) experienced disease relapse; in 8 of the 9 patients, disease relapsed in the retroperitoneum/paraaortic lymph-nodes. None of the 4 patients who received adjuvant chemotherapy developed relapsed tumors. All of the 9 men with disease relapse were treated with chemotherapy, and in 5 of them, surgical removal of tumors was performed. No patient had cause-specific death also in the nonseminoma group.

**Table 5:** Outcomes of patients with stage I nonseminomas

	**Surveillance**	**Chemotherapy**	**Total**
	**(n=36)**	**(n=4)**	**(n=40)**
Disease relapsed (n)	9 (25.0%)	0 (0%)	9 (22.5%)
Site			
Retroperitoneum	8 (88.9%)		
Mediastinum	1 (11.1%)		
Lung	1 (11.1%)		
Time to relapse (months)	2–13 (mean 6)		
Treatment at relapse (n)			
Chemotherapy	9		
Surgery	5		
Prognosis			
Cause-specific death	0	0	0
Death of other causes	1	1	2

**Figure 3:**
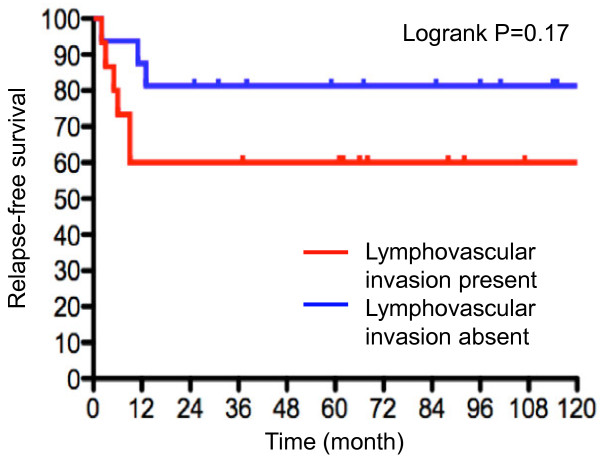
Impact of lymphovascular invasion on disease relapse-free survival in patients with stage I nonseminoma.

Both 5-year and 10-year relapse-free survival rates were 77.5% in the nonseminoma group, and they were 75.0% in men managed with surveillance. We further examined the influence of disease characteristics on relapse in patients with nonseminomas; the tumor size, pathological subtypes, tumor marker levels, or pT stage had no impact on disease relapse (Table 6). Regarding disease invasiveness based on histopathological examinations, patients with lymphovascular invasion seemingly had poorer relapse-free survival than those without; 40.0% and 18.8% of the patients with and without lymphovascular invasion had disease relapse, respectively, although the difference was not significant (*P*=0.17, Figure 3).

**Table 6:** Influence of disease characteristics on relapse in patients with nonseminomas

**Variables**	**No.**	**Relapse**	**Univariate analyses**		
		**n (%)**	**p**	**HR**	**95% CI**
					
Age <40	30	7 (23.3%)	0.92	1.08	0.23-5.21
≥40	10	2 (20.0%)		1	
Tumor size ≤5 cm	22	5 (22.7%)	0.88	0.90	0.21-3.75
>5 cm	12	3 (25.0%)		1	
Embryonal carcinoma No	11	4 (36.4%)	0.21	2.31	0.62-8.60
Yes	28	5 (17.9%)		1	
Choriocarcinoma No	32	8 (25.0%)	0.57	1.83	0.23-14.7
Yes	7	1 (14.3%)		1	
Teratoma No	17	3 (17.6%)	0.49	0.62	0.15-2.46
Yes	22	6 (27.3%)		1	
Yalk sac tumor No	19	3 (15.8%)	0.33	0.50	0.13-2.01
Yes	21	6 (28.6%)		1	
AFP Normal	8	2 (25.0%)	0.93	0.93	0.19-4.49
Elevated	29	7 (24.1%)		1	
βhCG Normal	13	4 (30.8%)	0.64	1.37	0.37-5.10
Elevated	23	5 (21.7%)		1	
LDH Normal	20	6 (30.0%)	0.49	1.63	0.41-6.51
Elevated	16	3 (18.8%)		1	
pT T1	16	3 (18.8%)	0.19	0.39	0.10-1.58
T2-4	15	6 (40.0%)		1	
Lymphovascular invasion No	16	3 (18.8%)	0.19	0.39	0.10-1.58
Yes	15	6 (40.0%)		1	

LDH, lactate dehydrogenase; AFP, α-fetoprotein, hCGβ, human chorionic gonadotropin β subunit.

## Discussion

Stage I testicular seminoma has been reported to relapse between 10% and 20% in previous studies [5,6], while adjuvant radiotherapy is frequently used with a relatively encouraging outcome as relapse rates of 3–4% [7]. In our institution, the overwhelming majority of men with stage I seminoma have recently been managed with surveillance (Table 3). In the current patient series managed with surveillance, the 10-year relapse-free survival rate reached 93.4%; it is speculated that diagnostic/staging modalities such as high-performance CT might lead to an appropriate exclusion of metastases and favorable outcomes. Also, none of them died during the observation period; the prognosis of men with stage I testicular seminoma is excellent when an appropriate surveillance protocol is applied. However, their postorchiectomy management remains a matter of concern, although adverse events including treatment-associated morbidity is less in adjuvant setting radiotherapy than in radical chemotherapy for clinically recurrent disease. A few previous studies tried to define risk-stratification of testis-confined seminoma. Aparicio and associates prospectively studied 314 men with stage I seminoma managed according to risk-adapted criteria. In their trial, those with tumor diameter less than 4 cm and no rete testis involvement were managed with surveillance; 6% of these patients still experienced relapse [8]. In our study, tumor burden, tumor markers, and pT were not associated with disease relapse. Concerning pathological characteristics, however, patients with disease extension through tunica albuginea showed a poorer relapse-free survival than those without. Although the difference was not significant (*P*=0.09, Figure 2), 3.8% of the patients without involvement of the tunica albuginea had relapse, whereas the relapse rate reached 20% in those with tunica albuginea involvement (Table 4). A recent retrospective study reported that tunica albuginea penetration was predictive of the presence of metastasis (n=86, *P*=0.00001), although the study recruited men with seminoma at all stages [9]. To verify its significance in risk-stratification of stage I seminoma, a high-volume study based on cancer registry is currently underway.

It also remains controversial how patients with stage I nonseminoma should be managed. Although cause-specific death was absent in our patient series, it has been fatal in 1% to 15% in previous reports [10-12]. Twenty-five to 30% of the patients with stage I nonseminoma managed by surveillance have been reported to experience disease relapse [7], and adjuvant chemotherapy has been the therapeutic standard for those with elevated tumor markers at diagnosis and/or highly malignant histopathology [8,11]. Vascular invasion and predominant embryonal carcinoma are generally considered to be histopathologic risk factors [7]. In our institution, men with stage I nonseminoma with normalized tumor markers or markers showing reductions assumed based on their half-life period are principally managed with surveillance regardless of the mentioned pathological characteristics, and the present study suggested that patients with lymphovascular invasion may have higher risk for relapse. Although the difference was not significant, 40.0% of the patients showing lymphovascular invasion had disease relapse, while 18.8% of those without it experienced relapse (Figure 3, Table 6).

The present study had several limitations. It was performed in a retrospective design, and the study volume was relatively small. Also, our database did not include information about the presence of some uncommon histological components such as sarcomatous differentiation coexistent with teratoma and potentially having an impact on oncological outcomes [4,13].

## Conclusions

In men with stage I seminoma, surveillance after orchiectomy is a feasible option. Although further studies are warranted, the present study suggested that tunica albuginea involvement may be a risk factor associated with disease relapse in them. In men with stage I nonseminoma and normalized markers after orchiectomy, surveillance is also a feasible option, but those with lymphovascular invasion may possibly be at high risk for disease relapse.

## Abbreviations

RFS: Relapse-Free Survival; CT: Computed Tomography; LDH: Lactate Dehydrogenase; AFP: α-fetoprotein; hCGβ: human Chorionic Gonadotropin β subunit.

## Competing interests

The authors declare that they have no competing interests.

## Authors’ contributions

KK conducted data analysis and helped to draft the manuscript. KK, YK, TS, TN, and NH performed surgical procedures and participated in clinical management of patients and data collection. TK performed histopathological diagnoses. NH wrote the manuscript and supervised throughout the study. KT assisted to draft the manuscript. Thank you for the assistance. All authors read and approved the final manuscript.
